# Multilocus genotyping reveals high heterogeneity and strong local population structure of the *Plasmodium vivax *population in the Peruvian Amazon

**DOI:** 10.1186/1475-2875-9-151

**Published:** 2010-06-03

**Authors:** Peter Van den Eede, Gert Van der Auwera, Christopher Delgado, Tine Huyse, Veronica E Soto-Calle, Dionicia Gamboa, Tanilu Grande, Hugo Rodriguez, Alejandro Llanos, Jozef Anné, Annette Erhart, Umberto D'Alessandro

**Affiliations:** 1 Department of Parasitology, Institute of Tropical Medicine, Antwerp, Belgium; 2 Instituto de Medicina Tropical Alexander Von Humboldt Universidad Peruana Cayetano Heredia, Lima, Peru; 3 Departamento de Bioquimica, Biologia Molecular y Farmacologia, Facultad de Ciencias, Universidad Peruana Cayetano Heredia, Lima, Peru; 4 Multi-Country Malaria Project "Malaria control on the cross border areas of the Andean Region: A community based approach"- PAMAFRO. Organismo Andinode Salud - Convenio Hipolito Unanue, Lima, Peru; 5 Catholic University of Leuven, Department Microbiology and Immunology, Leuven, Belgium

## Abstract

**Background:**

Peru is one of the Latin American countries with the highest malaria burden, mainly due to *Plasmodium vivax *infections. However, little is known about *P. vivax *transmission dynamics in the Peruvian Amazon, where most malaria cases occur. The genetic diversity and population structure of *P. vivax *isolates collected in different communities around Iquitos city, the capital of the Peruvian Amazon, was determined.

**Methods:**

*Plasmodium vivax *population structure was determined by multilocus genotyping with 16 microsatellites on 159 *P. vivax *infected blood samples (mono-infections) collected in four sites around Iquitos city. The population characteristics were assessed only in samples with monoclonal infections (n = 94), and the genetic diversity was determined by calculating the expected heterozygosity and allelic richness. Both linkage disequilibrium and the genetic differentiation (*θ*) were estimated.

**Results:**

The proportion of polyclonal infections varied substantially by site (11% - 70%), with the expected heterozygosity ranging between 0.44 and 0.69; no haplotypes were shared between the different populations. Linkage disequilibrium was present in all populations (*I*_A_^S ^0.14 - 0.61) but was higher in those with fewer polyclonal infections, suggesting inbreeding and a clonal population structure. Strong population differentiation (*θ *= 0.45) was found and the Bayesian inference cluster analysis identified six clusters based on distinctive allele frequencies.

**Conclusion:**

The *P. vivax *populations circulating in the Peruvian Amazon basin are genetically diverse, strongly differentiated and they have a low effective recombination rate. These results are in line with the low and clustered pattern of malaria transmission observed in the region around Iquitos city.

## Background

*Plasmodium vivax *is the major cause of human malaria outside the African continent, with an estimated annual burden of 70-80 million cases per year [1]. In the Americas, approximately 80% of the malaria cases reported in 2007 were due to *P. vivax *[2]. Peru is one of the Latin American countries with the highest malaria endemicity, with a negative impact on health and economic development. A large proportion of malaria cases (69% in 2006) occurs in the Amazonian department of Loreto with an uneven distribution, and with population migration patterns different from other Amazonian countries such as Brazil [3,4]. In Loreto, most malaria cases (82%) are caused by *P. vivax*, with an estimated incidence of 0.39 infections/person/malaria season [4]. To overcome adherence problems, the national drug policy has been shortened to 7 days of primaquine (PQ) at an increased daily dosage of 0.5 mg/kg/day combined with a 3-day chloroquine (CQ) (total 25 mg/kg) [5]. In this area, CQ resistant *P. vivax *has been recently reported [6].

Despite its importance, little information is available on *P. vivax *epidemiology, e.g. risk factors for infection, transmission dynamics and relapse rate, drug resistance and parasite population structure. Analysing the genetic diversity and the structure of the local parasite population in time and space provide new insights on the local distribution and dynamics of *P. vivax *transmission [7,8]. However, few studies have been conducted on the population structure of *P. vivax *in Latin America [9-11]. In settings like the Peruvian Amazon basin, where the malaria distribution is uneven and clustered [3,4,12,13], the *P. vivax *parasite population showed high diversity when genotyped using the Merozoite surface protein 3 alpha (MSP3α) marker alone [14]. Parasite population structure can also be analysed by microsatellites, simple sequence tandem repeats often used for studying the genetic diversity and population dynamics, such as those used in Brazil and Colombia where high heterogeneity and strong population structure was found [9-11]. In the present paper, the population structure of a set of *P. vivax *infected blood samples, collected around Iquitos city, the capital of Loreto Department, was analysed by multilocus genotyping.

## Methods

### Study sites and population (Figure 1)

The area around Iquitos is densely forested with many small pools, rivers and swamps near human settlements, offering ideal breeding sites for the main vector, *Anopheles darlingi*, a sylvatic species and very effective malaria vector [3,4]. Its biting activities occur near the breeding sites and throughout the night, with a peak between 6 pm and midnight [3]. The climate is tropical, warm and humid. Malaria transmission is perennial with a peak between November and May. *Plasmodium vivax *is responsible for about 82% of all infections and affects all age groups, with the highest prevalence in adults [3]. The population of the four study sites consists mainly of '*mestizos*', individuals that cannot be clearly identified as belonging to a specific ethnic minority. The main occupation in rural communities is subsistence farming (slash and burn agriculture in forest fields situated at easy walking or paddling distance), hunting, fishing and small-scale charcoal production. In urban and peri-urban areas, many people are jobless, or carry out occasional work (in fish farms, plantations, street sellers, etc...), are employees or shopkeepers.

**Figure 1:**
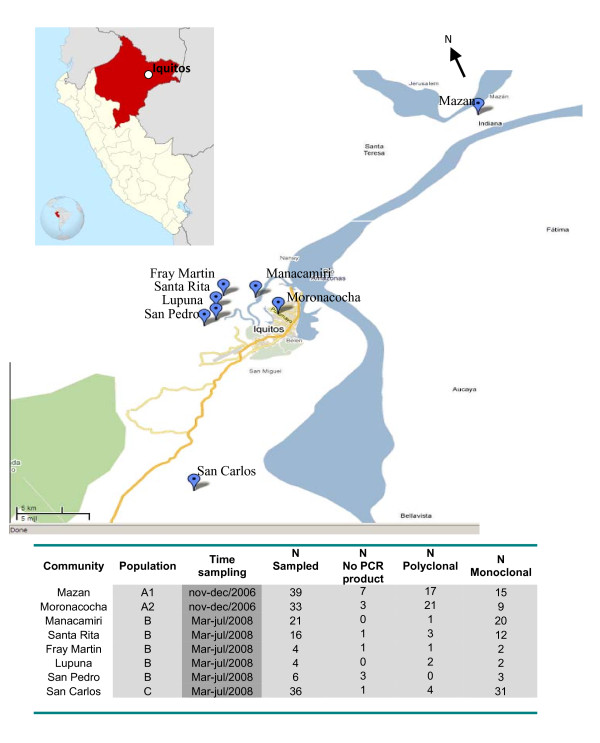
**Map of the study area (study sites mapped with GPS on Google maps) and number samples for each community (Total samples population = 159)**.

#### Mazan & Moronacocha in Iquitos North (Site A1 & A2)

Moronacocha health centre is situated in the Northern part of Iquitos city and covers a large range of urban and peri-urban communities (Figure 1). Patients are either resident in the nearby urban communities or from different parts of Loreto Department, on their way to the capital city. Mazan health centre is situated at about 20 km from Iquitos city and is accessible only by boat (45 min. from Iquitos, Figure 1). In Mazan jurisdiction, people mainly live in open or semi-open wooden houses on stilts and besides subsistence farming, practice also seasonal logging ('*madereros*') and consequently stay far out in the jungle for several weeks during the rainy season, where they usually sleep unprotected against mosquito bites. According to the local health staff, the local malaria incidence increases each year when 'madereros' return from the jungle (Erhart A., personal communication). Patients from Mazan may as well attend Moronacocha health centre where they pass by when going to Iquitos city. Both health centres are attended by people of neighbouring communities, but also by madereros coming from all over the Loreto Department.

#### Iquitos North, across the Rio Nanay (Site B)

This site includes five neighbouring villages (Manacamiri, Lupuna, Fray Martin, San Pedro and Santa Rita) located in the forest, at about 3-7 km north of Iquitos, on the other bank of the Rio Nanay, therefore only accessible by boat from Iquitos (Figure 1). There are two health posts, one in Manacamiri and the other in Lupuna covering the four remaining villages. Most people in Manacamiri live in closed, well-constructed houses while in Lupuna in semi-closed wooden houses on stilt. They practice mainly subsistence farming all year around, with occasional hunting and fishing. Unlike Site A, they do not practice logging and are occasionally employed in Iquitos city. This population is mainly sedentary, cultivating forest fields around their villages.

#### Iquitos South, San Carlos village (Site C)

San Carlos is a small and relatively isolated village situated in the jungle at about 24 km south-west of Iquitos (Figure 1). It is accessible either by foot (10 km from the main Iquitos - Nauta road) or by boat (6 h from Iquitos city) through the Itaya River. The population consists mainly of farmers practicing subsistence activities in and around their community, with occasional travels to the Iquitos market to sell and buy products.

### Sample collections

Between November and December 2006, in Mazan (A1) and Moronacocha (A2) health centres, systematic screening for malaria parasite was carried out in all patients consulting for fever or other malaria-related symptoms. This campaign was part of the malaria control activities implemented by the Multi-country Malaria Project PAMAFRO, a cross border malaria control initiative involving Peru, Ecuador, Colombia & Venezuela and funded by the Global Fund for HIV, TB and Malaria (GFHTM). Blood collection occurred before the mass screening campaign and insecticide-treated bed net distribution was carried out by the Ministry of Health in 2007. Between March and July 2008, systematic screening for the detection of malaria infections was carried out in Iquitos North, across the Rio Nanay (Site B) and in Iquitos South, San Carlos village (Site C).

After giving informed consent, explained in local language and including study objectives, procedures, risks and benefits, people were checked for malaria parasites by microscopy (thick and thin blood film) and a blood sample on filter paper (Whatman, grade 3, Whatman, Springfield Mill, USA) was collected for later genotyping. Quality control of microscopy was done blindly by a senior technician at the reference laboratory of the Regional Direction of Health in Iquitos (DIRESA). Microscopically confirmed malaria infections were treated according to the national treatment guidelines.

### Laboratory methods

Filter paper blood samples from patients with a microscopically confirmed *P. vivax *mono-infection were retrospectively selected for genotyping. DNA extraction was performed with the saponine-chelex method described elsewhere [15]. Samples were analysed by species-specific PCR [16] to confirm the *P. vivax *mono-infection, and then genotyped separately in 50 μL with 5 μL of DNA extract added to the reaction mix by using 17 microsatellites, 14 published in [17] and three in [18]. The final reaction conditions are described elsewhere [18]. The PCR product size was analysed on a 3730 XL ABI sequencer (Applied biosystems, Foster city, California, USA). Fragment sizes were determined with Genemapper (Applied Biosystems, Foster city, California, USA) using default microsatellite settings, whereby bands smaller than 100 relative fluorescence units (rfu) were defined as background [18]. Peaks above this threshold were considered as real alleles, except for MS16 because of stutter. Within each sample, only the peaks above 25% of the dominant one (highest rfu) were considered as real alleles [18].

### Data analysis

Data were entered and analysed in Excel (Microsoft cooperation, USA). An infection was defined as polyclonal if there was at least one locus that presented more than one allele. For each malaria infection, the locus with the highest number of alleles was considered as a proxy for the multiplicity of infection (MOI), representing the minimal number of parasite haplotypes in the sample. The MOI and the average number of alleles per locus were assessed for each of the 4 parasite populations, i.e. A1, A2, B and C.

The population characteristics were preferably assessed only in samples with monoclonal infections as the use of polyclonal infections might bias the calculation by generating artificial haplotypes [9-11]. The number of alleles/locus, the allelic richness, and the genetic diversity of each locus were computed for each population. Genetic diversity was assessed by calculating the expected heterozygosity (*He*) = [n/(n-1)][1-∑p_i_^2^], where n is the total number of alleles, p_i _is the frequency of the i^th ^allele in the population. The *He *represents the probability of finding a different allele for a given locus in any pair of haploids randomly drawn from the same population, and it was computed with FSTAT version 2.9.3 [19]. To evaluate the likelihood that identical haplotypes found in two or more samples originated from a different ancestry the p_sex _values were computed using GenClone ver. 2.0 [20].

The presence of overall multilocus linkage disequilibrium (LD = non random association of alleles occurring at different loci) was assessed with LIAN software version 3.5 [21]. The Standardized Index of Association (*I_A_^s^*) was estimated as a measure of linkage in each population, and the significance was tested using the Monte Carlo method. The presence of linkage disequilibrium was assessed in 1) the complete dataset, 2) monoclonal infections only, 3) unique haplotypes only, this for the overall dataset and in each population separately. When assessing linkage using the complete genotyping data, only the dominant alleles were considered.

Genetic differentiation *(θ) *was measured in FSTAT with the Cockerham & Weir method [19,22], first on the whole dataset and then by pair-wise comparison between populations. To test its significance, the obtained *θ *was compared with values obtained after random permutation (10,000 times) of multilocus genotypes between populations (not assuming Hardy-Weinberg equilibrium). Contingency tables of the genotypes by population were generated using the log-likelihood statistic, *G *[23]. Since the interpretation of the *θ *values from multi-allelic data can be affected by the level of diversity of the loci, the assessment of population differentiation requires standardization [24]. This was done following the method described by Meirmans [25], using the RECODEDATA software v.0.1 in FSTAT.

In addition to the population differentiation described with the *θ *values, the question on whether the same population structure applies when analyzing the samples according to their haplotypes was explored by evaluating the hypothetical population structure with the STRUCTURE v2.1 software [26]. This alternative Bayesian approach uses no *a priori *information and assigns samples to *K *populations based on the allele frequencies of each locus. The program was run 10 replicates with *K *varying from 1-10 with a burn-in period of 50,000 iterations followed by 150,000 iterations. The admixture model, allowing for mixed ancestry within individual isolates was applied. The most probable number of clusters was defined by calculating the Δ*K *value as described by Evanno *et al *[27].

## Results

### Genetic diversity

A total of 159 *P. vivax *mono-infections (39 and 33 in site A1 and A2, 51 in site B, and 36 in site C) were analysed (Figure 1). Only 16 polymorphic loci were retained for the analysis as locus Pv6727 was monomorphic in all samples. In 16 samples (seven from population A1, three from A2, five from B, and one from C), no PCR product was obtained in any of the 16 loci, despite the confirmation of *P. vivax *infection by species specific PCR. Therefore, the final analysis was carried out on 143 samples, i.e. 32 and 30 samples from population A1 and A2, respectively, 46 from population B and 35 from population C. Overall, 49 (34%) samples were polyclonal, occurring more frequently in population A1 (44%, n = 17) and A2 (70%, n = 21) than in population B (15% n = 7) and C (11% n = 4). The MOI was also higher in populations A1 and A2 (1.7 for both) than in B (1.2) and C (1.1). Nevertheless, the average number of alleles/locus and per sample (mean: 1.1) did not differ between the four sites.

Among the 94 monoclonal infections, population A1 and A2 displayed the highest genetic diversity (*He*) and allelic richness (Table 1). A total of 42 haplotypes could be identified (11 in A1, 7 in A2, 19 in B, and 5 in C), with none of the haplotypes shared between the four populations (Table 2). Within population B, haplotypes where shared between communities and between members of a single community. Within population C (single community), the haplotypes were shared between different households and household members. In all populations, the probability of having identical haplotypes derive from a different sexual reproduction was extremely small (p_sex _< 0.0001), suggesting the existence of a common ancestor and a clonal population structure as result of inbreeding.

**Table 1:** Number of alleles and genetic diversity (*He*) by locus and population (N = 94 monoclonal infections).

	Number of Alleles/locus				Allelic Richness				*He*			
	
Populations	A1	A2	B	C	A1	A2	B	C				
	n = 15	n = 9	n = 39	n = 31	h = 11	h = 7	h = 19	h = 5	A1	A2	B	C
MS1	**4**	**3**	**3**	**2**	3.96	2.99	2.15	1.45	**0.77**	**0.56**	**0.2**	**0.07**
MS2	**5**	**3**	**4**	**4**	4.74	2.99	3.33	3.16	**0.79**	**0.67**	**0.65**	**0.57**
MS3	**3**	**3**	**3**	**2**	2.96	2.99	2.99	1.86	**0.59**	**0.42**	**0.67**	**0.19**
MS4	**5**	**4**	**3**	**3**	4.62	3.99	2.73	2.71	**0.73**	**0.81**	**0.36**	**0.55**
MS5	**2**	**2**	**2**	**2**	1.96	1.99	1.96	1.45	**0.25**	**0.22**	**0.27**	**0.07**
MS6	**6**	**3**	**5**	**5**	5.51	2.99	4.1	3.61	**0.83**	**0.67**	**0.64**	**0.59**
MS7	**3**	**2**	**1**	**2**	2.58	2	1	2	**0.26**	**0.56**	**0**	**0.43**
MS8	**5**	**5**	**9**	**3**	4.55	5	5.73	2.45	**0.73**	**0.89**	**0.74**	**0.47**
MS9	**5**	**4**	**6**	**4**	4.34	3.99	4.81	3.3	**0.56**	**0.69**	**0.74**	**0.59**
MS10	**6**	**3**	**4**	**2**	5.34	2.99	3.46	1.71	**0.8**	**0.42**	**0.53**	**0.13**
MS12	**6**	**5**	**7**	**3**	5.68	4.97	4.71	2.45	**0.85**	**0.72**	**0.64**	**0.47**
MS15	**6**	**6**	**6**	**4**	5.5	5.97	3.86	3.16	**0.81**	**0.89**	**0.51**	**0.57**
MS16	**8**	**6**	**7**	**4**	7.25	5.97	4.91	3.3	**0.91**	**0.83**	**0.71**	**0.59**
MS20	**6**	**6**	**5**	**4**	5.47	5.97	4.44	3.41	**0.77**	**0.89**	**0.75**	**0.59**
Pv6635	**5**	**4**	**6**	**4**	4.34	3.99	4.75	3.3	**0.56**	**0.69**	**0.72**	**0.59**
Pvsal	**6**	**6**	**5**	**5**	5.16	5.97	4.45	3.61	**0.74**	**0.89**	**0.69**	**0.59**

**Mean**	**4.81**	**4.44**	**4.75**	**3.31**	4.62	4.05	3.71	2.64	**0.66**	**0.69**	**0.55**	**0.44**

h: number of unique haplotypes

**Table 2:** Haplotypes detected by patient and population.

Patient	Haplotype	Profile	Population	SV	SHH
					
PAT 20	H1	adccbbacdfcfkadh	A2		
PAT 8	H2	baabbdacabhbbean	A1		
PAT 53	H3	baacbeacefcdfffe	B		
PAT 55	H4	baacbeajefcfbffe	B	1	0
PAT 60	H4	baacbeajefcfbffe	B	1	0
PAT 68	H5	baacbealeccfbffl	B		
PAT 22	H6	baacbgcefchdedgm	C	1	0
PAT 23	H6	baacbgcefchdedgm	C	1	0
PAT 24	H6	baacbgcefchdedgm	C	1	0
PAT 25	H6	baacbgcefchdedgm	C	1	0
PAT 28	H6	baacbgcefchdedgm	C	1	0
PAT 30	H6	baacbgcefchdedgm	C	1	0
PAT 39	H6	baacbgcefchdedgm	C	1	0
PAT 40	H6	baacbgcefchdedgm	C	1	0
PAT 50	H6	baacbgcefchdedgm	C	1	0
PAT 13	H7	baaeadagdfcfbcei	A1		
PAT 54	H8	baccbeajeccfbhfl	B		
PAT 65	H9	badcbeacefcdfffe	B		
PAT 62	H10	badcbeaiefdgbhfe	B		
PAT 57	H11	badcbeajefcfbffe	B		
PAT 61	H12	badcbeajefcfbhfe	B	0	0
PAT 69	H12	badcbeajefcfbhfe	B	1	0
PAT 79	H12	badcbeajefcfbhfe	B	1	0
PAT 64	H13	badcbgaiiaddbfjj	B		
PAT 26	H14	bcadbhaceccenjfa	C	1	1
PAT 27	H14	bcadbhaceccenjfa	C	1	1
PAT 29	H14	bcadbhaceccenjfa	C	1	0
PAT 31	H14	bcadbhaceccenjfa	C	1	0
PAT 32	H14	bcadbhaceccenjfa	C	1	1
PAT 33	H14	bcadbhaceccenjfa	C	1	1
PAT 34	H14	bcadbhaceccenjfa	C	1	1
PAT 35	H14	bcadbhaceccenjfa	C	1	1
PAT 36	H14	bcadbhaceccenjfa	C	1	1
PAT 37	H14	bcadbhaceccenjfa	C	1	1
PAT 38	H14	bcadbhaceccenjfa	C	1	0
PAT 41	H14	bcadbhaceccenjfa	C	1	1
PAT 42	H14	bcadbhaceccenjfa	C	1	1
PAT 43	H14	bcadbhaceccenjfa	C	1	1
PAT 44	H14	bcadbhaceccenjfa	C	1	0
PAT 46	H14	bcadbhaceccenjfa	C	1	0
PAT 49	H14	bcadbhaceccenjfa	C	1	0
PAT 15	H15	bdabcdacachbicab	A2		
PAT 16	H16	bdafbdagchgdaccj	A2		
PAT 21	H17	bdafbgahacebfeal	A2		
PAT 52	H18	bdccbaacdfcfkbeh	B	1	0
PAT 56	H18	bdccbaacdfcfkbeh	B	1	0
PAT 73	H18	bdccbaacdfcfkbeh	B	1	0
PAT 78	H18	bdccbaacdfcfkbeh	B	1	0
PAT 47	H19	bdccbaacdgcfkbeg	C	1	1
PAT 48	H19	bdccbaacdgcfkbeg	C	1	1
PAT 71	H20	bdccbaajefcfbhfh	B		
PAT 45	H21	beabbfachcedkdid	C		
PAT 76	H22	beabbfadhcedmein	B		
PAT 63	H23	beacbeacdfafkbee	B	0	0
PAT 70	H23	beacbeacdfafkbee	B	0	0
PAT 66	H24	beacbeajifaffhje	B		
PAT 18	H25	beadbgceccccabcf	A2	1	0
PAT 19	H24	beadbgceccccabcf	A2	1	0
PAT 67	H26	beaeadaahchdddin	B		
PAT 11	H27	beaeagacheacaghj	A1		
PAT 77	H28	bebcbeajefafbhfe	B		
PAT 75	H29	bedcbdabbdgaghbj	B		
PAT 72	H30	bedeadakbdgfbdbj	B	0	0
PAT 74	H30	bedeadakbdgfbdbj	B	0	0
PAT 6	H31	bedfbhaeadaafgaj	A1		
PAT 12	H32	bfccbibaccdekkcn	A1		
PAT 10	H33	ccabbhacaccdleae	A1		
PAT 7	H34	cccbbhafabdbkcan	A1		
PAT 14	H35	dbabbdagccaehhcb	A2		
PAT 4	H36	dccabeacacbdjfal	A1		
PAT 3	H37	dedcbgceccbfkdch	A1		
PAT 17	H38	deebbgcafccalife	A2		
PAT 51	H39	eacbacaddccbmdek	C		
PAT 58	H40	eacbadaddccbmfel	B	1	0
PAT 59	H40	eacbadaddccbmfel	B	1	0
PAT 5	H41	ebacbjaeaagcacaj	A1		
PAT 1	H42	ebacbjaeaagcccaj	A1	1	0
PAT 2	H42	ebacbjaeaagcccaj	A1	1	0
PAT 9	H42	ebacbjaeaagcccaj	A1	1	0

For each marker, the alleles were ranked by molecular size and then labeled from a to × (Profile). Profile reports the different alleles for each marker in a single patient. Haplotypes found in the same village (SV) or the same house hold (SHH) are indicated as present (1) or absent(0).

### Linkage disequilibrium

Linkage disequilibrium was assessed first on all infections (polyclonal and monoclonal) and then only on monoclonal infections. Significant linkage disequilibrium (p < 0.0001) was found in the whole dataset (n = 94), and was most prominent in population C, while the lowest *I_A_^s ^*was obtained for A2 and then A1 (Table 3). When repeating the analysis using only the 42 unique haplotypes, the linkage disequilibrium remained significant except for population C, where there may have been an epidemic expansion. In population C, the vast majority of monoclonal infections (83.9%, 26/31) were due to only two out of the total five haplotypes present, which were markedly different from each other.

**Table 3:** Linkage disequilibrium (standardized index of association, *I*_A_^S^) by *P. vivax *population and overall.

Population	Monoclonal infection *I*_A_^S^	Unique haplotypes *I*_A_^S^	All infections *I*_A_^S^
A1	0.277*	0.097*	0.137*
A2	0.144*	0.025*	0.086*
B	0.206*	0.199*	0.246*
C	0.610*	-0.023	0.561*
**TOTAL**	0.177*	0.09*	0.172*

*Significant, α = 0.05.

### Population differentiation

The overall standardized *θ *for the whole dataset was 0.45 (p = 0.0001), indicating a strongly structured population with little migration between sites. Pair-wise analysis revealed that the four populations could be divided into three clusters, one comprising populations A1 and A2, and the other two corresponding to population B and C, respectively, with the latter being the most differentiated from the remaining populations (Table 3). The difference observed between population A1 and A2 was not significant. The high number of identical multi-locus genotypes, especially in population C, could have possibly inflated the *θ *values. Therefore the analysis was repeated using only the unique haplotypes (n = 42). Although the *θ *values decreased by half, the relative differences between the populations were maintained and no significant genetic differentiation was observed between populations A1 and A2. Furthermore, no genetic differentiation between communities within clusters was found (Table 4).

**Table 4:** *θ *values obtained by pair-wise analysis on 94 monoclonal samples.

	PopA1	PopA2	PopB						PopC
				*B1*	*B2*	*B3*	*B4*	*B5*	
**PopA1**		**0.29**	0.51*	0.55*	***0.26***	***0.29***	***0.19***	0.55*	0.60*
**PopA2**	0.09		0.52*	0.57*	***0.38***	***0.22***	***0.15***	0.58*	0.50*
**PopB**	0.20*	0.21							0.58*
***B1***	0.23*	0.25*			***0.27***	***0.38***	***0.21***	***0.05***	0.59*
***B2***	*0.08*	*0.12*		*0.12*		***-1.75***	***-0.56***	***-0.22***	**0.75**
***B3***	*0.09*	*0.07*		*0.17*	*-0.47*		***-0.61***	***0.17***	**0.62**
***B4***	*0.06*	*0.05*		*0.09*	*-0.16*	*-0.16*		***0.19***	**0.48**
***B5***	0.22*	0.24*		*0.02*	*-0.10*	*0.07*	*0.08*		0.66*
**PopC**	0.28*	0.24*	0.29*	0.32*	*0.40*	*0.32*	*0.24*	0.35*	

*Significant at the 5% level following Bonferroni correction (0.8%) for multiple testing.

**Numbers in bold **in the upper triangle are standardized *θ *values, the others being the uncorrected *θ *values. B1 = Manacamiri, B2 = Lupuna, B3 = Fray Martin, B4 = San Pedro, B5 = Santa Rita.

**Figure 2:**
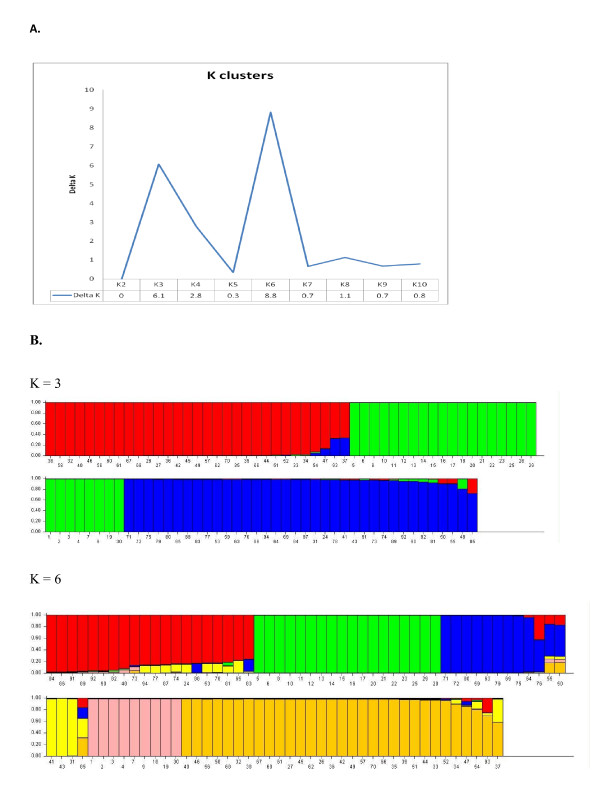
**Estimated *P. vivax *population structure by STRUCTURE software**. **Panel A **represents the STRUCTURE results for the whole dataset, showing two peaks at K = 3 and 6 (delta K = 6 and 8.8). **Panel B **contains the bar plot at K = 3 and K = 6 with each sample being represented by a single vertical line divided into K colors, where K is the number of populations assumed. Each color represents one population, and the length of the colored segment shows the estimated proportion of membership of that sample to each population. Sample number 1-31 belong to population C, 32-70 to population B, 71-85 to population A1, and 86-94 to population A2.

When using STRUCTURE, a primary peak of Δ*K *(Δ*K *= 6) was observed for K = 3 (Figure 2), confirming the existence of the three clusters. However, the highest Δ*K *value (Δ*K *= 8.8) was obtained for K = 6. Larger subgroups were mainly found in populations C and A1 while in population B only a minor group was split off from the larger cluster containing the majority of samples.

## Discussion

In this study, the four *P. vivax *populations were highly heterogeneous with varying degrees of polyclonal infections (11% to 70%), genetic diversity (0.44 to 0.69), and linkage disequilibrium (*I*_A_^S ^from 0.144 to 0.610). Similarly to other populations of both *P. falciparum *and *P. vivax*, linkage disequilibrium was higher in populations with fewer polyclonal infections, indicating increased inbreeding and a clonal population structure [10,28]. Within this dataset no haplotypes were shared among the populations. Moreover, there was strong differentiation between populations, except for A1 and A2, indicating little exchange between sites. Strong genetic differentiation has been frequently observed between *P. vivax *population in Latin America where the malaria transmission appears to be highly clustered [3,4,9-13]. However, it should be noted that in the present study the sampling has been done at different periods, at the end of 2006 for Mazan (population A1) and Moronacocha (population A2), and in 2008 for the other 2 sites, population B and C. Between sample collections in A1 and A2 and those in B and C, the Peruvian malaria control program launched an intensive campaign of active detection and treatment of malaria infections and distribution of insecticide-treated bed nets. The campaign resulted in a 40% decrease of malaria incidence in Loreto [29] and might have reduced the parasite diversity and the percentage of polyclonal infections, possibly resulting in increased inbreeding and hence a more clonal population structure as observed in population B compared to A1 and A2. However, no intervention was carried out in San Carlos (population C) and in Santa Rita, one community within population B. Therefore, the similarities in diversity, haplotypes and MOI between Santa Rita and the other communities in population B could be explained by the little impact that control efforts had on the parasite diversity. However, given that the villages surrounding Santa Rita were included in the control programme, it may also affected Santa Rita, as population B can be considered as one population. In addition, the number of samples may have been too low to detect an impact on the different communities in population B. Possibly, the different methodology used for collecting samples in 2006 (passive detection, covering a large area where the *madereros *work) and 2008 (active, local sampling) could partly explain the higher diversity and genetic differentiation in populations A1 and A2 as compared to B and C. Nevertheless, the differences observed between the populations can also be explained by the heterogeneity of malaria transmission and the difference in human population movements [3,4,12,13]. Indeed, around Iquitos the *P. vivax *populations were highly structured, with the largest differences between geographically isolated sites. The strong population differentiation between populations A1&A2 and the two other sites (*θ*_standardized _between 0.5 - 0.6), particularly for population C, indicates little exchange between populations despite their geographical proximity. Indeed, people from site B and C spend most of their activities in and around their communities and seldom visit or travel to other places. Conversely, the lack of genetic differentiation and the shared haplotypes among communities located within site B is an indication of regular contact between neighbouring communities. This was supported by the epidemiological data. Despite the small number of samples available for each community, a limitation for detecting any inter-community genetic differentiation, some samples of population B were found in different clusters when the analysis was done by STRUCTURE.

No major genetic differentiation between A1 (Mazan) and A2 (Moronacocha) was found, even though they were located at about 25 km from each other, a larger distance than that between A2 and the area corresponding to population B. Both health centres drain patients from various and overlapping areas and therefore lack major genetic differentiation that can be explained by the human population movements occurring between both cities, as these are important trading centres, especially for *madereros*, increasing the probability of parasite mixing.

San Carlos (population C) is a geographically isolated site with no direct access to the main road and little contact with other communities. Bayesian analysis revealed further clustering of population C in two sub-groups, probably the result of its clonal population structure. This was reflected by the high frequency of *P. vivax *infections carrying one of the two dominant haplotypes and the absence of linkage disequilibrium when only the unique haplotypes were considered, suggesting a recent epidemic expansion of those two clones [28,30]. However, the number of unique haplotypes might have been too small to detect low levels of linkage disequilibrium although the five haplotypes found were very distinct from each other. The hypothesis of a local epidemic expansion was also supported by epidemiological data, i.e. the number of malaria cases per year was usually very low before increasing substantially, for unknown reason, at the beginning of 2008 (Veronica E. Soto-Calle, personal communication).

Linkage disequilibrium was present also in populations A1 and A2, where the proportion of polyclonal infections was high and remained so when increasing the samples size by including the polyclonal samples. The common occurrence of inbreeding despite the high polyclonality has already been observed in other *P. vivax *population in Brazil and Colombia [8-10,31], even though its causes remain unknown. The occurrence of *P. vivax *asymptomatic infections is common in the area around Iquitos (Veronica E. Soto-Calle, personal communication) and has already been reported in neighbouring Amazonian countries [32]. Therefore, polyclonal infections may result from the accumulation of parasite clones from different episodes (relapses, new infections) that remain untreated. Partial immunity could alter the densities of viable gametocytes in a strain specific manner, negatively influencing the infectivity to mosquitoes and increasing the probability of in-breeding despite the polyclonality [33]. Alternatively, linkage disequilibrium might result from the local population sub-structuring, the so-called Wahlund effect [30,34]. The population admixture may lead to linkage disequilibrium between physically unlinked loci, causing false-positive linkage signals. Indeed, the health centres of Mazan and Moronacocha drain patients originating from different areas in Loreto, with the possibility that populations A1 and A2 include two or more parasite sub-groups. Despite the limited sample size for each population, analysis with STRUCTURE confirmed the presence of further sub-structuring, as observed for population A1 and B.

## Conclusions

The *P. vivax *population in Iquitos and neighbouring areas is diverse and highly structured, with strong genetic differentiation between geographically isolated sites, suggesting that transmission is extremely local and confined within each cluster [3,4]. These findings were comparable to those reported from Brazil where the *P. vivax *and *P. falciparum *parasite populations were spatially and temporally clustered [9,10,35-37]. Although both species are believed to be transmitted by the same vector (*A. darlingi*), the local *P. vivax *population in Peru and Brazil was more diverse than *P. falciparum *[14,37]. The presence of hypnozoites enables the parasite to bridge periods of low transmission and possibly increases the *P. vivax *population diversity. Little is known about the complex local dynamics of *P. vivax *strains and the importance of relapses on the *P. vivax *epidemiology and population structure. Further investigations on the geographical population subdivision and temporal dynamics within a cohort of people prospectively followed up are needed to better understand such dynamics, especially before and after adequate treatment.

## Conflict of interests

The authors declare that they have no competing interests.

## Authors' contributions

PVDE carried out the PCR analysis, data analysis and paper writing. GVDA and CD assisted with the analysis. GVA and TH helped data analysis and review of the article. AE and UDA made substantial contributions to conceive the study design, paper writing and reviewing. VSC, DG, AL, TG and HR coordinated the sampling and field studies in Peru. JA revised the manuscript. All authors read and approved the final manuscript
